# Temporal dynamics and demographic specificity of provider-initiated HIV testing in China: an eight-year retrospective analysis

**DOI:** 10.3389/fpubh.2025.1616709

**Published:** 2025-11-26

**Authors:** Yuexin Sun, Qiaofang Wu, Yue Tao, Haoyue Yu, Ying Zhou, Jun Bao

**Affiliations:** Department of Dermotology, Nanjing Drum Tower Hospital, Affiliated Hospital of Medical School, Nanjing University, Nanjing, Jiangsu, China

**Keywords:** HIV testing, provider-initiated testing and counseling (PITC), positivity rates, retrospective analysis, epidemiological trends

## Abstract

**Background:**

This study evaluates the effectiveness of PITC implementation at Nanjing Drum Tower Hospital from 2017 to 2024 and examines variations in testing efficiency across different populations and time periods, aiming to provide critical evidence for adjusting PITC strategies to adapt to epidemiological changes.

**Methods:**

A cross-sectional analysis study was conducted on 907,395 patients who underwent HIV testing at Nanjing Drum Tower Hospital from 2017 to 2024. Patients were divided into routine testing group (RT group) (*n* = 879,503) and PITC group (*n* = 27,892). Chi-square tests and Cochran-Armitage trend tests were employed to compare HIV positivity rates and temporal trends between the two groups.

**Results:**

The overall HIV positivity rate in the PITC group (0.51%) was significantly higher than in the RT group (0.10%) (*χ*^2^ = 718.5, *p* < 0.001), although the effect size was modest (*V* = 0.028). Time trend analysis revealed a significant decline in the positivity rate within the PITC group, from 0.83% in 2017 to 0.30% in 2024 (*Z* = −2.96, *p* = 0.003). In contrast, the positivity rate in the RT group remained relatively stable (0.07–0.13%). The PITC group was predominantly male (67.5%), largely composed of young adults aged 20–40 years (61.8%), with unmarried individuals accounting for 63%. Conversely, the RT group consisted mainly of middle-aged and older adults married women (58.2%), with a higher proportion undergoing first-time testing (72.5%). Notably, the HIV positive rate in the PITC group dropped significantly in 2023 and 2024, reaching 0.20 and 0.30% respectively, potentially due to the “dilution effect” following an expansion of the testing population or issues related to data quality.

**Conclusion:**

PITC demonstrated significant improvements in HIV testing efficiency during 2017–2022.

## 1 Introduction

The underutilization of human immunodeficiency virus (HIV) testing services poses a significant barrier to the effective implementation of HIV prevention and treatment strategies (1, 2). Early detection of HIV facilitates timely access to medical care and antiretroviral therapy, thereby improving survival rates and potentially reducing HIV transmission (3). According to the 2025 World Health Organization report, HIV remains a major global public health challenge (4). In 2024, approximately 1.3 million new HIV infections globally occurred worldwide, representing a continued decline from previous years due to effective prevention strategies and expanded access to testing and treatment (5). However, certain groups in China continue to experience an alarming increase in AIDS infection rates (6).

Traditional HIV counselling and testing has historically been provided in two primary settings. The first setting is patient-initiated voluntary counseling and HIV Testing (VCT), where individuals proactively opt for HIV testing. The second setting involves routine health screenings prior to surgery, prenatal care, and blood transfusions. Recognizing the limitations of these conventional approaches, a third method has emerged. Since May 2007, the World Health Organization (WHO) and UNAIDS have officially endorsed PITC (7). This provider-driven model encourages patients to undergo HIV testing and counseling and is recommended for “all adults and adolescents seen in all health facilities” in regions with an HIV prevalence exceeding 1%, provided that adequate prevention, treatment, and care resources are available.

However, the implementation of PITC in primary hospitals and local clinics still faces challenges such as insufficient testing facilities and shortages of human resources (8). More importantly, the current promotion of PITC lacks sufficient localized evidence on HIV positivity rates to support its effectiveness (9). Existing studies have shown that PITC is particularly effective in high-prevalence areas. For example, a study in Benue State, Nigeria, evaluated the acceptability and feasibility of PITC among adolescents and adults in secondary healthcare centers. Compared to voluntary counseling and testing (VCT), PITC increased the number of people tested for HIV by 5% and demonstrated good acceptability and feasibility (10). However, the efficiency of PITC is influenced by various factors, including gender, lower education levels, type of testing, and the department where the test is conducted. Moreover, findings vary across different regions (11, 12). To date, most studies have focused on high-prevalence areas or specialized clinics (such as STD or TB clinics), while data from low-prevalence settings or general outpatient departments remain limited (13). Although international research has confirmed the value of PITC, direct comparative data between PITC and traditional testing methods remain scarce in Asian healthcare settings. Existing studies often focus on specific populations (e.g., MSM) (14), leaving the difference in detection rates among the general patient population unclear. To address this gap, we conducted a retrospective study analyzing patients who underwent routine HIV testing and PITC at Nanjing Drum Tower Hospital from 2017 to 2024. We compared HIV positivity rates under different testing strategies and conducted a preliminary exploration of the public health impact of PITC, measured by differences in HIV positivity rates and demographic patterns between PITC and RT strategies. This study aims to provide stronger evidence to support the broader implementation of PITC.

## 2 Subjects and methods

### 2.1 Clinical information

**Figure 1:**
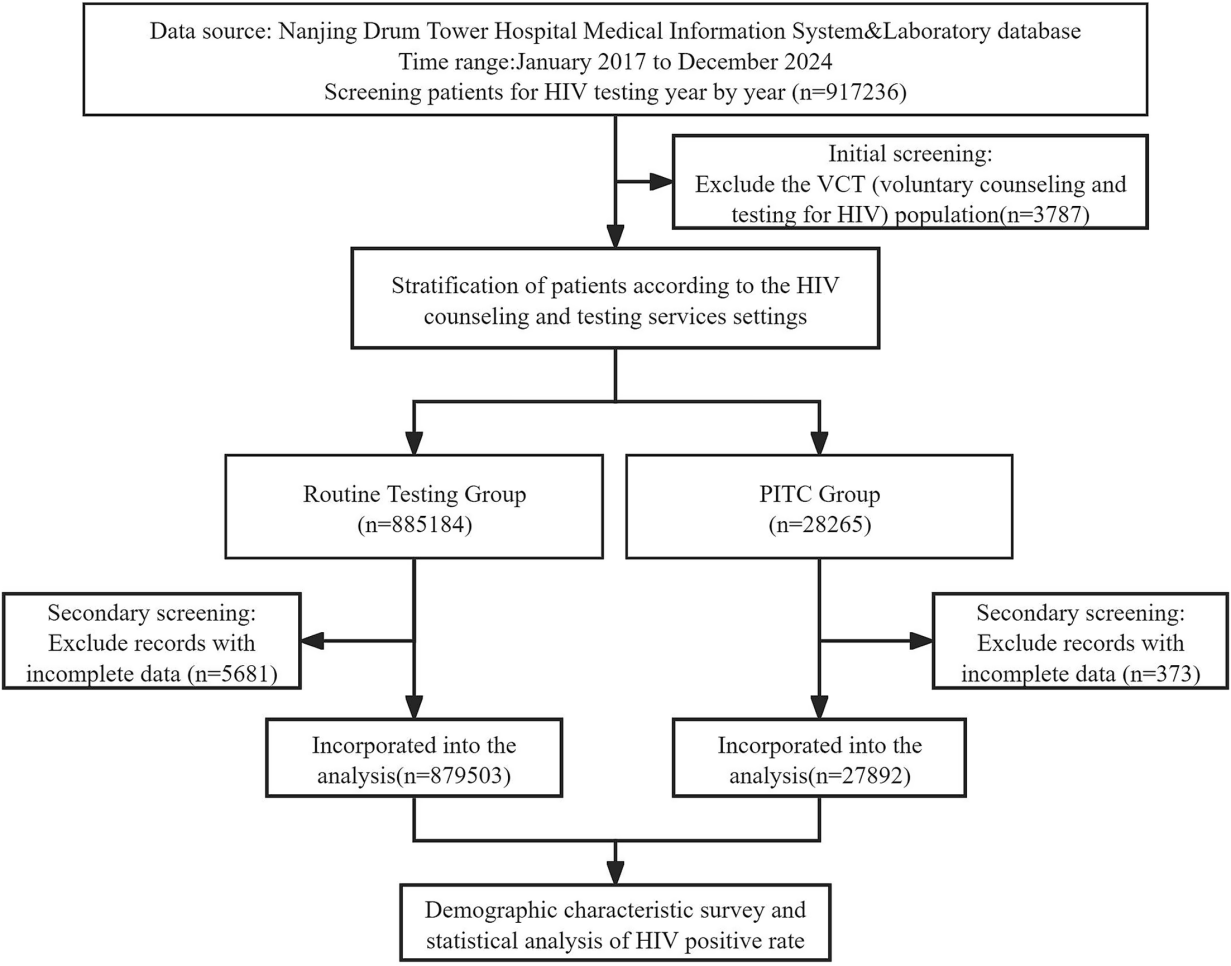
Technical roadmap of this study.

This was a retrospective concurrent-control study based on routinely collected hospital and clinical laboratory data from 2017 to 2024, comparing HIV testing outcomes between provider-initiated testing (PITC) and routine testing (RT). We divided the research subjects into two main groups: the routine HIV testing group (RT group), which included patients before surgery, those undergoing invasive examinations or requiring blood transfusions, as well as those undergoing premarital physical examinations and pregnant women; and the PITC group, which referred to HIV testing proactively recommended by medical staff based on the patients’ specific conditions, mainly targeting individuals with high-risk behaviors (including non-marital heterosexual sex, men who have sex with men [MSM], intravenous drug users [IDU], spouses or children of HIV-infected individuals, and others), those with relevant clinical symptoms, and those with a history of sexually transmitted infections (STIs). The research process is shown in Figure 1.

### 2.2 Methods

By searching the medical information system and clinical laboratory database, we identified patients who underwent HIV testing at Nanjing Gulou Hospital from January 2017 to December 2024. The HIV test kits adopted by our hospital are HISCL HIV Ag + Ab Assay Kit (SYSMEX CORPORATION). These patients were categorized into two groups based on different HIV testing methods: the PITC group and the RT group. The HIV positivity rates between the two groups were compared, and demographic characteristics of the patients in each group were analyzed. Epidemiological data, including gender, age, marital status, and previous detection history, were retrospectively reviewed. Through one-to-one responsibility physicians visits and consultation, relevant information of patients who underwent PITC testing was collected, including whether there is a high risk behavior and attitude toward HIV testing and PITC. High-risk behavior classification includes heterosexual sex of non-marital, MSM, IDU, HIV-infected person or the patient’s spouse or children and others. The primary outcome measure was the HIV test result (positive or negative).

### 2.3 Statistical analysis

All data analyses were conducted using SPSS software (version 20.0, IBM SPSS Statistics, Chicago, IL). Continuous data were expressed as mean±standard deviation (x̄±s), categorical data were presented as number (percentage). Inter-group comparisons were conducted using *χ*^2^ tests. Given the exceptionally large sample size (*n* = 907,395), all statistical tests yielded significant results (*p* < 0.001). Therefore, the practical significance should be assessed in conjunction with the effect size. The interpretation of the effect size (Cramer’s V) is as follows: 0.1 indicates a weak association, 0.3 suggests a moderate association, and 0.5 denotes a strong association. For intra-group comparisons, chi-square goodness-of-fit tests were conducted to assess whether the observed distribution within groups significantly deviated from the theoretical uniform distribution. Cochran-Armitage trend test was used for time trend analysis for intra-group comparisons in Table 1. A *p* value < 0.05 was considered statistically significant.

**Table 1:** Positive population and proportion of HIV tests in the RT and PITC groups.

Year	RT Group			PITC Group			*χ*^2^	P^*^
	Negative	Positive	Positive proportion (%)	Negative	Positive	Positive proportion (%)		
2017	75,265	76	0.10%	836	7	0.83%	55.2	<0.01
2018	80,532	87	0.11%	975	11	1.12%	99.1	<0.01
2019	96,940	71	0.07%	1,022	9	0.87%	124.5	<0.01
2020	101,176	89	0.09%	2,763	24	0.86%	128.5	<0.01
2021	130,996	165	0.13%	3,212	23	0.71%	86.3	<0.01
2022	118,461	143	0.12%	4,978	33	0.66%	83.7	<0.01
2023	132,853	128	0.10%	7,315	15	0.20%	6.1	<0.05
2024	142,411	110	0.08%	6,649	20	0.30%	29.2	<0.01
Total	878,634	869	0.10%	27,750	142	0.51%	718.5	<0.05

^*^Inter-group comparisons (RT Group vs. PITC Group) were conducted using the chi-square test and Fisher’s exact test (for small sample sizes).

## 3 Results

In this study, we compared population characteristics and HIV positivity rates between the RT group (*n* = 879,503, 96.9%) and the PITC group (*n* = 27,892, 3.1%).

Demographic characteristics of the RT and PITC groups are presented in Table 2. Inter-group comparison analysis revealed significant differences in demographic and testing characteristics between the PITC and RT groups. Specifically, the proportion of males was significantly higher in the PITC group compared to the RT group (67.5% vs. 41.8%, *χ*^2^ = 176,907, *p* < 0.001; Cramer’s *V* = 0.44), indicating a strong association. Regarding age distribution, the PITC group had a higher proportion of individuals aged under 20 years (13.4% vs. 6.0%) and those aged 20–30 years (28.0% vs. 20.8%), while the proportions of individuals aged 40–50 years (16.4% vs. 25.7%) and over 50 years (8.3% vs. 16.2%) were lower (*χ*^2^ = 54,193, *p* < 0.001; Cramer’s *V* = 0.25). From the perspective of marital status, the proportion of unmarried individuals in the PITC group was significantly higher than that in the RT group (63.0% vs. 43.8%, *χ*^2^ = 44,800, *p* < 0.001; Cramer’s *V* = 0.22).

**Table 2:** Population characteristics in the RT and PITC groups.

Items	RT group	PITC group	Total	*χ*^2^	*P*	Cramer’s V
*n* (%)	879,503 (96.9)	27,892 (3.1)	907,395 (100)			
Sex, *n* (%)						
Male	367,857 (41.8)	18,829 (67.5)	386,686 (42.6)	176,907	<0.001	0.44^**^
Female	511,646 (58.2)	9,063 (32.5)	520,709 (57.4)			
Age category, *n* (%)						
<20	52,770 (6.0)	3,741 (13.4)	56,511 (6.2)	54,193	<0.001	0.25^*^
20–29	182,789 (20.8)	7,813 (28.0)	190,602 (21.0)			
30–39	275,496 (31.3)	9,435 (33.8)	284,931 (31.4)			
40–49	226,260 (25.7)	4,578 (16.4)	230,838 (25.4)			
>50	142,188 (16.2)	2,325 (8.3)	144,513 (15.9)			
Marital status, *n* (%)						
Married	493,890 (56.2)	10,329 (37.0)	504,219 (55.6)	44,800	<0.001	0.22^*^
Unmarried	385,613 (43.8)	17,563 (63.0)	403,176 (44.4)			
Test type, *n* (%)				84,651	<0.001	0.31^*^
Repeat test in ≤12 months	111,829 (12.7)	6,378 (24.2)	118,567 (13.1)			
Repeat test in>12 months	130,283 (14.8)	5,341 (19.1)	135,624 (14.9)			
Never tested	637,391 (72.5)	15,813 (56.7)	653,204 (72.0)			

^*^Moderate association; ^**^Strong association.

Intra-group comparisons indicated that the RT group was predominantly female (58.2% vs. 41.8%, *χ*^2^ = 46,134.5, *p* < 0.001), with a concentration of individuals in the 30–40 age group (31.3%), significantly deviating from a uniform distribution (*χ*^2^ = 1,020,176, *p* < 0.001). In contrast, the PITC group showed an imbalanced distribution of gender (67.5% male) and marital status (63.0% unmarried, both *p* < 0.001), with a higher concentration of individuals in the 30–40 age group (33.8%, *χ*^2^ = 6,241.6, *p* < 0.001). The majority of patients in the PITC group had never been tested (56.7%), but the proportion of repeat tests (43.3%) was significantly higher than expected under a uniform distribution assumption (*χ*^2^ = 7,182.8, *p* < 0.001).

In Table 3, we compared demographic characteristics, testing behaviors, and risk factors between HIV-positive and HIV-negative individuals within the PITC cohort using Chi-square tests and Cramer’s V to quantify effect sizes. Regarding demographic characteristics, the proportion of individuals aged 20–30 years in the HIV-positive group was significantly higher than in the HIV-negative group (35.9% vs. 27.8%, *χ*^2^ = 8.45, *p* = 0.038), while the proportion of those aged 30–40 years was lower (26.8% vs. 33.7%). The proportion of individuals who are unmarried and in a relationship is significantly higher among those who are HIV positive compared to those who are HIV negative (62.7% vs. 47.4%, *χ*^2^ = 31.52, *p* < 0.001). Moreover, the proportion of HIV-positive individuals in the “Unmarried, in a relationship” group is much higher than in other groups. In terms of testing behavior, the proportion of first-time testers in the HIV-positive group is significantly higher than that of repeat testers (79.6% [113/142] vs. 20.4% [29/142], *χ*^2^ = 58.21, *p* < 0.001). Additionally, the HIV positivity rate in never tested group is 0.71%. Concerning high-risk behaviors and health awareness, intravenous drug use was more prevalent in the HIV-positive group (4.2% vs. 0.0%, *p* < 0.001), although this finding should be interpreted cautiously due to the small sample size. Additionally, HIV awareness was significantly lower in the HIV-positive group compared to the HIV-negative group (34.5% vs. 7.1%, *χ*^2^ = 97.34, *p* < 0.001). While the overall effect size between groups were weak (Cramer’s *V* = 0.017–0.059), the actual percentage differences were substantial. In the HIV-negative group, the majority were female (65.5% vs. 34.5%, *χ*^2^ = 12,345.6, *p* < 0.001), with most individuals falling into the 30–40 age group (33.7%) and predominantly engaging in no prior testing (56.6%). In contrast, the HIV-positive group exhibited a higher prevalence of intravenous drug use (4.2%) and history of HIV spouse/child contact (6.3%), significantly deviating from the distribution observed in the HIV-negative group. Among HIV-positive individuals, the majority (79.6%) were first-time testers, and only a small proportion had previously undergone HIV testing.

**Table 3:** Population characteristics by HIV status in the PITC groups.

Characteristics	HIV test result			*χ*^2^	*P*	Cramer’s V
	Negative	Positive	Total			
*n* (%)	27,750 (99.5)	142 (0.5)	27,892 (100.0)			
Sex, *n* (%)						
Male	18,742 (67.2)	87 (61.3)	18,829 (67.5)	1.12	0.29	0.006
Female	9,008 (32.3)	55 (38.7)	9,063 (32.5)			
Age category, *n* (%)						
<20	3,724 (13.4)	17 (12.0)	3,741 (13.4)	8.45	0.038	0.017
20–29	7,762 (27.8)	51 (35.9)	7,813 (28.0)			
30–39	9,397 (33.7)	38 (26.8)	9,435 (33.8)			
40–49	4,555 (16.3)	23 (16.2)	4,578 (16.4)			
>50	2,312 (8.3)	13 (9.2)	2,325 (8.3)			
Marital status, *n* (%)						
Married	10,301 (37.1)	28 (19.7)	10,329 (37.0)	31.52	<0.001	0.034
Unmarried, in a relationship	13,148 (47.4)	89 (62.7)	13,237 (47.4)			
Separated or widowed	4,148 (14.9)	19 (13.4)	4,167 (14.9)			
No sexual partners	153 (0.6)	6 (4.2)	159 (0.6)			
Test type, *n* (%)						
Repeat test in≤12 months	6,731 (24.3)	7 (4.9)	6,738 (24.2)	58.21	<0.001	0.045
Repeat test in>12 months	5,319 (19.2)	22 (15.5)	5,341 (19.7)			
Never tested	15,700 (56.6)	113 (79.6)	15,813 (56.7)			
High-risk behavior classification, *n* (%)						
Heterosexual sex of non-marital	16,638 (60.0)	83 (58.5)	16,721 (59.9)	59.87	<0.001	0.046
MSM (men who have sex with men)	7,931 (28.6)	37 (26.1)	7,968 (28.6)			
IDU (intravenous drug users)	3 (0.0)	6 (4.2)	9 (0.0)			
HIV infected person or the patient’s spouse or children	1,308 (4.7)	9 (6.3)	1,317 (4.7)			
Others	1,870 (6.7)	7 (4.9)	1,877 (6.7)			
Attitudes toward PITC and HIV testing, *n* (%)						
Little or no knowledge of HIV and testing, *n* (%)	1,976 (7.1)	8 (5.6)	1,984 (7.1)	97.34	<0.001	0.059
Personally favorable toward PITC, *n* (%)	25,475 (91.8)	134 (94.4)	25,609 (91.8)			

Table 1 presents a comparative analysis of HIV testing data between the RT Group and the PITC Group from 2017 to 2024. The study reveals a statistically significant difference in HIV test positivity rates between the two groups, although the effect size is modest. Specifically, the overall positivity rate for the PITC group (0.51%) was significantly higher than that of the RT group (0.10%) (*χ*^2^ = 718.5, *p* < 0.001), indicating that the PITC strategy may be more effective in identifying potential HIV infections. However, the effect size (Cramer’s *V* = 0.028) suggests that the practical significance of this difference is limited, potentially due to the substantial disparity in testing volumes (879,000 tests in the RT group versus 28,000 in the PITC group).

Annual comparisons show that from 2017 to 2022, the PITC group consistently exhibited higher positivity rates (ranging from 0.66 to 1.12%) compared to the RT group (0.07 to 0.13%), with all differences being statistically significant (*p* < 0.001). However, from 2023 to 2024, the positivity rate in the PITC group declined markedly (to 0.20–0.30%), narrowing the gap with the RT group (0.08–0.10%), though still remaining statistically significant (*p* = 0.014–0.001). Time trend analysis indicates a significant downward trend in the PITC group’s positivity rate (Cochran-Armitage trend test: *Z* = −2.96, *p* = 0.003), decreasing from 0.83% in 2017 to 0.30% in 2024. The peak positivity rate was observed in 2018 at 1.12%, followed by a gradual decline. Notably, after 2023, there was a sharp drop in the PITC group’s positivity rate, which may be attributed to adjustments in local HIV prevention and control policies.

In contrast, the RT group showed no significant time trend (*Z* = −1.90, *p* = 0.057), with positivity rates fluctuating between 0.07 and 0.13%, peaking at 0.13% in 2021 and reaching its lowest point of 0.07% in 2019.

Within-group annual differences were also analyzed. For the PITC group, the annual variability in positivity rates was significant (*χ*^2^ = 28.3, *p* < 0.001), suggesting that changes in the target population or intervention measures may have influenced testing efficiency. Despite a substantial increase in testing volume from 2,763 in 2020 to 6,649 in 2024, the positivity rate concurrently decreased, possibly reflecting a “dilution effect” as the coverage expanded. In the RT group, while annual differences were also significant (*χ*^2^ = 45.8, *p* < 0.001), the fluctuations were relatively minor (0.07–0.13%), indicating greater stability in its testing model.

## 4 Discussion

The present study provides critical insights into the evolving efficacy of PITC at Nanjing Drum Tower Hospital from 2017 to 2024. While PITC demonstrated superior HIV detection rates compared to routine testing overall, its performance exhibited notable temporal fluctuations and demographic-specific variations. These findings underscore both the promise and challenges of PITC implementation in dynamic epidemiological settings.

The PITC group initially achieved a significantly higher HIV positivity rate (0.51% vs. 0.10% in routine testing), aligning with studies from Mozambique and Kenya where targeted PITC strategies improved case detection (9, 15, 16). However, the marked decline in PITC positivity from 1.12% (2018) to 0.30% (2024) (*Z* = −2.96, *p* = 0.003) raises critical questions. Under the national-level strategic execution of informatization planning, China has gradually built the world’s largest direct reporting system for infectious disease outbreaks and public health emergencies since 2016, which has optimized the PITC process and improved testing efficiency. Additionally, at the end of 2019, the Jiangsu Provincial Health Commission issued the implementation plan to curb the spread of HIV/AIDS, aiming to maximize the identification and treatment of HIV-infected individuals. This may have driven the substantial increase in PITC testing volumes starting in 2020. However, this trend may also lead to the “dilution effect”: since 2020, the expansion of the PITC testing population has diluted the focus on high-risk subgroups. Alternatively, improved pre-test risk stratification in later years could have reduced unnecessary testing, inadvertently missing marginal-risk cases. Similar declines were observed in Sub-Saharan African countries’ PITC programs when coverage expanded without proportional increases in high-risk targeting (17, 18).

The analysis of demographic characteristics reveals that the PITC group is characterized by younger, unmarried males with a higher likelihood of repeat testing, whereas the conventional testing group includes more middle-aged and older adults females who are married. The strong association between PITC and younger, unmarried males (Cramer’s *V* = 0.44 for gender) highlights its success in reaching populations with documented high-risk behaviors (19, 20). However, the weak overall effect size (*V* = 0.028) for positivity rate differences suggests that PITC’s advantage is context-dependent. While PITC detected 5.1-fold more cases than routine testing in 2017–2022, the narrowing gap in 2023–2024 (0.20–0.30% vs. 0.08–0.10%) underscores the need for adaptive strategies. This mirrors findings from Mozambique, where periodic recalibration of PITC criteria was essential to maintain detection efficiency (15). It should be noted that the population differences between the RT and PITC groups reflect the inherent characteristics of the testing strategies, rather than a flaw in the study design. Future studies may consider using stratified sampling to improve comparability between the two groups in terms of age and gender.

Jiangsu Province’s 5.9% year-on-year decline in new HIV cases (2024) coincides with PITC’s expanded implementation but contrasts with rising infections among youth and seniors (21). Our data suggest that PITC’s declining yield may reflect shifting risk profiles—older adults and females, underrepresented in PITC cohorts, now constitute growing at-risk populations. Integrating age-stratified PITC protocols and expanding provider-initiated testing to geriatric and maternal health clinics could address this gap, as proposed in recent Mozambique, northern Tanzania and Cameroon guidelines (15, 21, 22).

Overall, the period from 2017 to 2022 appears to have been a critical phase for the PITC strategy, during which it effectively targeted high-risk populations such as young unmarried individuals and intravenous drug users. Future research should focus on optimizing the PITC strategy by refining target population selection, particularly by examining characteristics of high-risk years like 2018, while enhancing data quality monitoring during periods of lower positivity rates. The findings suggest that while the PITC strategy demonstrated an efficiency advantage during a specific timeframe, recent fluctuations highlight the need for dynamic adjustments in target population identification and continuous data tracking to improve the precision of HIV testing.

Despite PITC’s advantages—higher acceptance rates (91.8% in our cohort) and streamlined care pathways—its implementation faces systemic hurdles. The 2023–2024 positivity drop may signal testing fatigue or stigma-driven avoidance among high-risk groups, exacerbated by insufficient counselor training. The programs’ success in America, Mozambique and Malawi mitigated similar issues through community-led destigmatization campaigns and mobile testing units (23–25). We propose three optimizations. Primarily, implement dynamic target management by real-time surveillance to refine PITC eligibility criteria (e.g., integrating geospatial risk mapping). Moreover, conduct rigorous quality audits on anomalous data, specifically investing the unusual 0.20% positivity rate in 2023 to identify potential data gaps or protocol deviations. Lastly, establish cross-departmental collaboration. Partner with non-governmental organization (NGO) to reach underserved groups (e.g., rural MSM, older adults widows) excluded from hospital-based PITC (26).

This study’s concurrent control design minimized temporal confounders, offering robust evidence of PITC’s conditional superiority. However, this study has several limitations. Firstly, regarding the sample size, the PITC group constitutes 3.1% of the total sample, which may limit the statistical power to detect subtle risk factors; future studies should oversample high-risk subgroups. Secondly, in terms of unmeasured confounders, marital status and testing history alone cannot fully capture risk heterogeneity; integrating behavioral surveys would enhance precision. Due to the difficulty of investigating detailed individual risk factors in the RT group in clinical practice, it is challenging in this study to use propensity score methods or to accurately compare HIV detection rates between subgroups with similar baseline characteristics. Last but not least, concerning the data currency, projections to 2024 require validation against finalized surveillance reports.

## 5 Conclusion

While PITC remains a potent tool for HIV case identification, its efficacy is non-static. The strategy’s early success in targeting young, high-risk males must evolve to address Jiangsu’s emerging epidemiological trends. Sustaining PITC’s impact demands adaptive targeting, quality assurance, and cross-sector partnerships. Future research should employ mixed methods to disentangle programmatic, behavioral, and data quality drivers of the observed decline in PITC yield.

## Data Availability

The original contributions presented in the study are included in the article/supplementary material, further inquiries can be directed to the corresponding authors.
